# Local Complement Contributes to Pathogenic Activation of Lung Endothelial Cells in SARS-CoV-2 Infection

**DOI:** 10.1165/rcmb.2022-0373OC

**Published:** 2023-04-18

**Authors:** Hui Zhang, Evgenia Gerasimovskaya, Mary K. McCarthy, Nicholas A. May, Ram Raj Prasad, Suzette Riddle, B. Alexandre McKeon, Sushil Kumar, Min Li, Cheng-Jun Hu, Maria G. Frid, Thomas E. Morrison, Kurt R. Stenmark

**Affiliations:** ^1^ Cardiovascular Pulmonary Research Laboratories, Department of Pediatrics and Medicine,; ^2^ Department of Immunology and Microbiology, and; ^3^ Department of Craniofacial Biology, School of Medicine, University of Colorado Anschutz Medical Campus, Aurora, Colorado

**Keywords:** coronavirus disease (COVID-19), lung microvascular endothelial cells, hypoxia, extracellular ATP, inflammation

## Abstract

Endothelial dysfunction and inflammation contribute to the vascular pathology of coronavirus disease (COVID-19). However, emerging evidence does not support direct infection of endothelial or other vascular wall cells, and thus inflammation may be better explained as a secondary response to epithelial cell infection. In this study, we sought to determine whether lung endothelial or other resident vascular cells are susceptible to productive severe acute respiratory syndrome coronavirus 2 (SARS-CoV-2) infection and how local complement activation contributes to endothelial dysfunction and inflammation in response to hypoxia and SARS-CoV-2–infected lung alveolar epithelial cells. We found that ACE2 (angiotensin-converting enzyme 2) and TMPRSS2 (transmembrane serine protease 2) mRNA expression in lung vascular cells, including primary human lung microvascular endothelial cells (HLMVECs), pericytes, smooth muscle cells, and fibroblasts, was 20- to 90-fold lower compared with primary human alveolar epithelial type II cells. Consistently, we found that HLMVECs and other resident vascular cells were not susceptible to productive SARS-CoV-2 infection under either normoxic or hypoxic conditions. However, viral uptake without replication (abortive infection) was observed in HLMVECs when exposed to conditioned medium from SARS-CoV-2–infected human ACE2 stably transfected A549 epithelial cells. Furthermore, we demonstrated that exposure of HLMVECs to conditioned medium from SARS-CoV-2–infected human ACE2 stably transfected A549 epithelial cells and hypoxia resulted in upregulation of inflammatory factors such as ICAM-1 (intercellular adhesion molecule 1), VCAM-1 (vascular cell adhesion molecule 1), and IL-6 (interleukin 6) as well as complement components such as C3 (complement C3), C3AR1 (complement C3a receptor 1), C1QA (complement C1q A chain), and CFB (complement factor B). Taken together, our data support a model in which lung endothelial and vascular dysfunction during COVID-19 involves the activation of complement and inflammatory signaling and does not involve productive viral infection of endothelial cells.

**Clinical Relevance**

Emerging evidence does not support direct infection of lung endothelial or other vascular wall cells in coronavirus disease (COVID-19). Herein, we show that lung endothelial and vascular dysfunction in COVID-19 involves the activation of complement and inflammatory signaling and does not require productive viral infection of endothelial cells. The results of our study suggest that therapeutic strategies directed at strengthening the endothelial barrier, eliminating vascular inflammation, and complement activation could be helpful in ameliorating endothelial injury and cardiovascular complications in patients with COVID-19.

Coronavirus disease (COVID-19) caused by severe acute respiratory syndrome coronavirus 2 (SARS-CoV-2) infection is largely characterized by a progressive lung disease, in which lung alveolar epithelial and inflammatory cells are the primary targets of viral invasion. However, clinical data and autopsies on COVID-19 cases have demonstrated that COVID-19 can be a multisystem inflammatory disease characterized by intravascular coagulation with underlying endothelial dysfunction, indicating vascular involvement in its pathogenesis (1, 2). Clinical autopsy reports have also demonstrated that thrombotic microangiopathy, vasculitis, and intussusceptive angiogenesis in the lungs can be observed in COVID-19 cases (3).

Although endothelial cell (EC) dysfunction and inflammation play an essential role in the vascular pathology of COVID-19 (1), there are conflicting reports as to whether ECs or other resident vascular cells are susceptible to direct SARS-CoV-2 infection (4, 5). ACE2 (angiotensin-converting enzyme 2), which mediates SARS-CoV-2 cellular entry, is highly expressed in airway epithelial cells and also at much lower concentrations in other cell types, including vascular and cardiac cells (6, 7). Viral entry into the cell is facilitated by cell surface proteases, including TMPRSS2 (transmembrane serine protease 2), the liposomal cysteine proteases cathepsins B and L, and other factors, such as neuropilin-1 and basigin (CD147) (4, 8, 9). Previous studies suggested that low susceptibility of ECs to SARS-CoV-2 infection may be due to negligible expression of ACE2 (10). However, there is conflicting evidence regarding the correlation of ACE2 expression and the susceptibility of vascular endothelium to SARS-CoV-2 infection (10–12). SARS-CoV-2 may cause a form of abortive infection of EC without productive virus replication (13). Additional evidence suggests that SARS-CoV-2 may trigger NLRP3 (NLR family pyrin domain containing 3) inflammasome and caspase-1–mediated cytokine release and pyroptosis in several cell types (14, 15). However, at present, the complex pathological responses of ECs to SARS-CoV-2 are not fully understood.

SARS-CoV-2 infection is often accompanied by significant alveolar hypoxia, which can contribute to hyperinflammatory responses in the lungs and epithelial–endothelial interface. Under these conditions, pulmonary vascular ECs can be activated by cytokines, chemokines, and other inflammatory mediators, including nucleotides (e.g., ATP, ADP) released from damaged and activated epithelial and immune cells (1, 16), indicating that indirect EC activation may occur during SARS-CoV-2 infection. The pathological vascular endothelial responses in the lungs are augmented by hypoxia, which can contribute to endothelial hyperpermeability, inflammatory cell recruitment, and metabolic changes in vascular and immune cells (1, 17). However, whether or how hypoxia may potentiate, either directly or indirectly, pathological EC responses to SARS-CoV-2 remains unknown.

The complement system is an important component of the innate immune system and has been shown to play a role in the pathological remodeling of the vascular wall (18). Deregulated complement activation may fuel cytokine-driven hyperinflammation and thrombotic microangiopathy, thereby leading to multiorgan failure (19–21). Prominent activation of complement pathways, including the lectin (MASP-2 [MBL-associated serine protease 2]), classical, alternative, and terminal pathways, has been observed in the lungs, skin, and sera of SARS-CoV-2–infected individuals, suggesting that the complement system is a pathological trigger of COVID-19 (20, 21). Complement deposition on ECs and high C5a (complement C5a) serum concentrations have been linked to complement activation leading to systemic thrombotic microangiopathy (22). In turn, inhibition of C3 and C5 complement components ameliorated disease-associated hyperinflammation and endothelial damage (23, 24). Other studies have suggested direct involvement of the complement system in immune cell pyroptosis, increased endothelial permeability, and vascular dysfunction (25), supporting the idea that SARS-CoV-2 infection may directly or indirectly activate the complement system in vascular cells (26).

Previous work in our laboratory demonstrated that activation of the complement cascade (as demonstrated by C3d deposition) was consistently observed in a perivascular-specific manner in human pulmonary arterial hypertension (PAH) and animal models of PAH (27). However, it remains unknown if SARS-CoV-2 infection can synergize with hypoxia and activate the local complement system in the lungs and contribute to endothelial inflammation and dysfunction. In this study, we found that human lung microvascular ECs (HLMVECs) are not susceptible to productive SARS-CoV-2 infection under either control or hypoxic conditions. However, viral uptake without replication (abortive infection) was observed. Our study also demonstrated that exposure of HLMVECs to conditioned medium of SARS-CoV-2–infected human ACE2 stably transfected A549 (hACE2-A549) cells resulted in inflammatory and complement activation responses that were, to various extents, potentiated by hypoxia. Taken together, our data support previous findings that lung endothelial and vascular dysfunction in COVID-19 involves the activation of complement and inflammatory signaling and does not involve productive viral infection of ECs.

## Methods

Methods are described in detail in the data supplement.

### Viruses

SARS-CoV-2 strain 2019 n-CoV/USA_WA1/2020 was obtained from BEI Resources. The virus was passaged once in Vero E6 cells and titrated using a focus formation assay (FFA) on Vero E6 cells. The recombinant icSARS-CoV-2-mNeonGreen (mNG), also USA_WA1/2020 (28), was kindly provided by Dr. Pei-Yong Shi (University of Texas Medical Branch). The presence of an intact furin cleavage site was sequence confirmed in all virus stocks.

### Cells

Vero E6 cells were obtained from American Type Culture Collection (CRL-1586). hACE2-A549 cells were obtained from Dr. Mario Santiago at the University of Colorado School of Medicine (29). Cells were grown in a 5% CO_2_ atmosphere at 37 °C in Dulbecco’s modified Eagle medium containing 10% FBS, 100 U/ml penicillin, and 100 ng/ml streptomycin for Vero E6 cells or puromycin (0.5 μg/ml) for hACE2-A549 cells. Detailed cell culture, treatment procedures, and all assays on primary HLMVECs, human lung pericytes, human lung fibroblasts, and human distal pulmonary artery (dPA) smooth muscle cells (SMCs) are described in the data supplement.

### FFA

Infectious virus in cell culture supernatants was quantified using an FFA, as described in the data supplement.

### Generation and Use of hACE2-A549 Conditioned Medium

hACE2-A549 cells were plated at 2 × 105 cells per well in a 6-well plate. The following day, cells were mock inoculated or inoculated with SARS-CoV-2 diluted in PBS supplemented with 1% FBS and Ca^2+^/Mg^2+^ at a multiplicity of infection (MOI) of 0.5 fluorescence-forming units (FFU)/cell. Virus was absorbed for 1 hour at 37 °C, cell monolayers were washed with PBS, and 2 ml fresh culture medium was added to each well. At 24 and 48 hours postinfection (hpi), cell culture supernatants were collected, clarified by centrifugation at 800 × *g*, and stored at −80 °C. For stimulation of cell cultures, growth medium was removed, 250 μl conditioned medium from mock-infected or SARS-CoV-2–infected cells was added for 1 hour at 37 °C, followed by the addition of normal cell growth medium.

### Flow Cytometry

Flow cytometry for mNG reporter assay is described in the data supplement.

### qRT-PCR Analysis

All primer sets for real-time RT-PCR are listed in Table E1 in the data supplement. Results are presented as expression relative to control group or hypoxanthine phosphoribosyl transferase using the delta threshold cycle method. Approaches to the quantification of viral RNA are described in the data supplement.

### Western Blot Analysis

Western blot analysis was performed with specific antibodies for target proteins and is described in the data supplement.

### Statistical Analysis

Values are expressed as mean ± SEM. Prism 8.0 (GraphPad Software Inc.) was used to determine significance. The unpaired Student’s *t* test was used to compare two groups. For more than two groups with two independent variables, two-way ANOVA followed by Tukey’s multiple-comparisons test was performed. The Kolmogorov-Smirnov, Shapiro-Wilk, and D’Agostino tests were used to assess for normality before applying parametric statistical tests. Nonparametric testing was performed if data did not pass the parametric assumption. Differences with *P* values <0.05 were considered statistically significant.

## Results

### Comparative Analysis of ACE2 and TMPRSS2 mRNA Concentrations among Different Lung Vascular Cell Types

**Figure 1.**
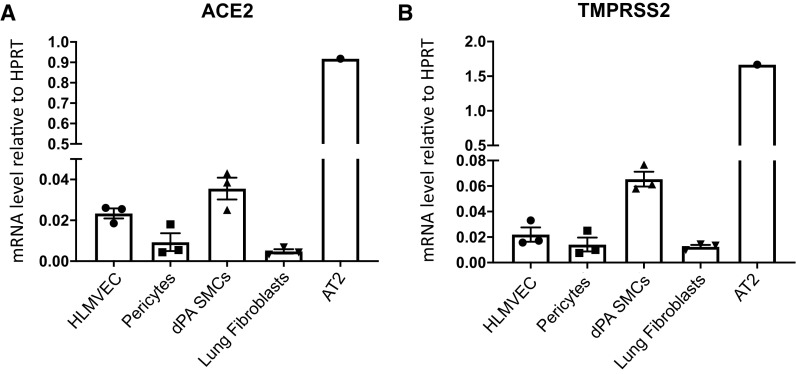
ACE2 (angiotensin-converting enzyme 2) and TMPRSS2 (transmembrane serine protease 2) are differentially expressed among different lung vascular cell types. (*A* and *B*) Total RNA was isolated from human HLMVECs, pericytes, dPA SMCs, lung fibroblasts, and AT2 cells (cultured in normal medium), and mRNA concentrations of ACE2 (*A*) and TMPRSS2 (*B*) were determined using qRT-PCR analysis. Data are presented as mean ± SEM. AT2 = alveolar epithelial type II; dPA = distal pulmonary artery; HLMVEC = human lung microvascular endothelial cell; HPRT = hypoxanthine phosphoribosyl transferase; SMC = smooth muscle cell.

ACE2 and serine protease TMPRSS2 provide SARS-CoV-2 spike protein binding and priming to promote viral cell entry (30). To begin to assess the susceptibility of primary human lung vascular cells to SARS-CoV-2 infection, we determined ACE2 and TMPRSS2 mRNA expression among different vascular cell types (in normal culture medium), including HLMVECs, pericytes, dPA SMCs, lung fibroblasts, and alveolar epithelial type II cells for comparison. qRT-PCR analysis revealed that although all vascular cell types express ACE2 and TMPRSS2, the expression amount was 20- to 90-fold lower compared with alveolar epithelial type II cells (Figure 1), a cell type known to express ACE2 and TMPRSS2 to support SARS-CoV-2 infection (4, 6, 10).

### Hypoxia and Extracellular ATP Differentially Regulate ACE2 and TMPRSS2 Expression in HLMVECs, Pericytes, SMCs, and Fibroblasts

**Figure 2.**
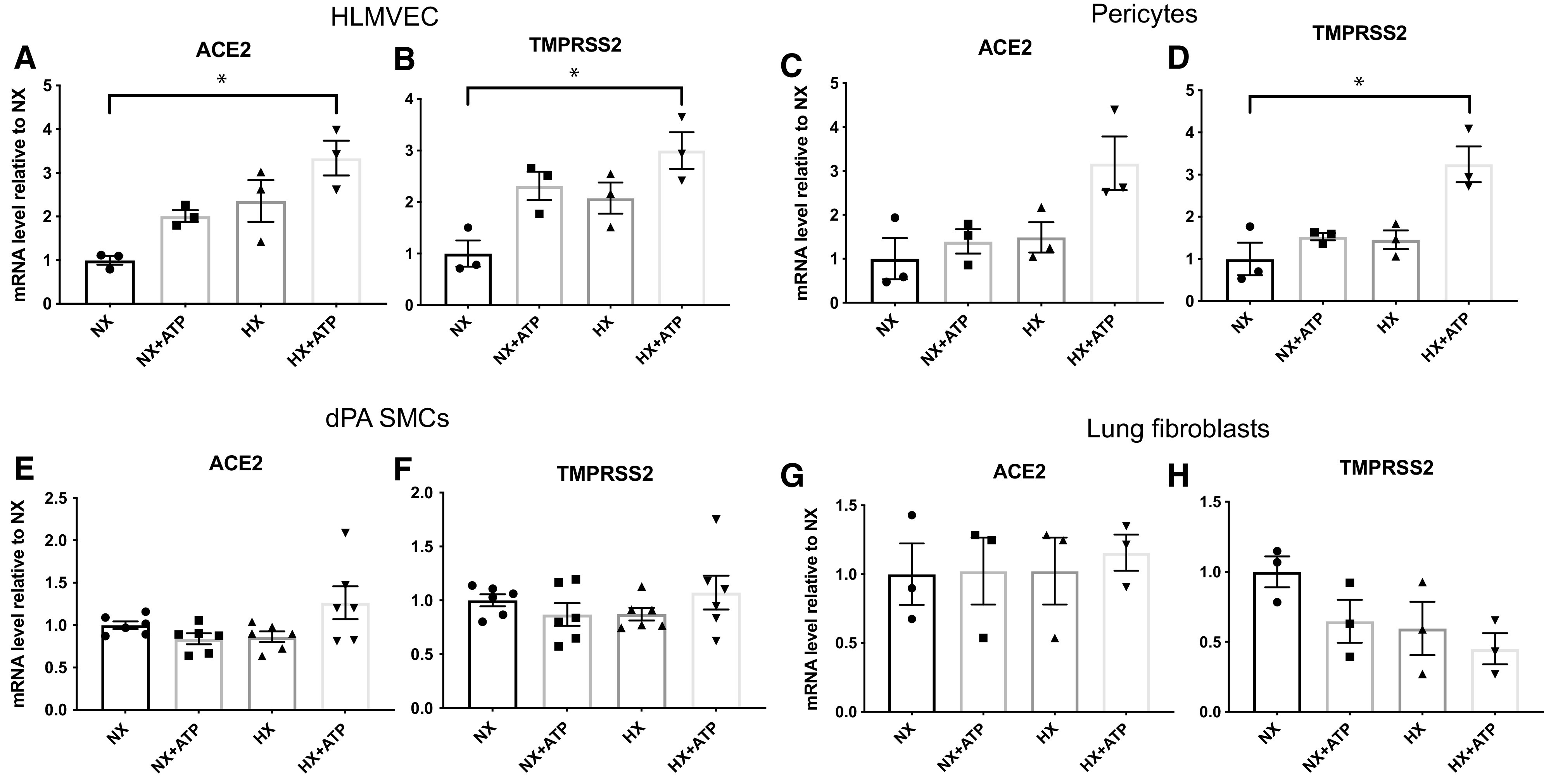
Hypoxia (HX) and extracellular ATP upregulate ACE2 and TMPRSS2 mRNA concentrations in HLMVECs but do not affect ACE2 and TMPRSS2 mRNA concentrations in SMCs and lung fibroblasts. Cells were cultured in normal medium and exposed to HX (3% O_2_) or extracellular ATP (100 μM) for 24 hours or remained untreated, and mRNA concentrations of ACE2 and TMPRSS2 were determined using qRT-PCR analysis. (*A* and *B*) ACE2 (*A*) and TMPRSS2 (*B*) concentrations in HLMVECs. (*C* and *D*) ACE2 (*C*) and TMPRSS2 (*D*) concentrations in pericytes. (*E* and *F*) ACE2 (*E*) and TMPRSS2 (*F*) concentrations in dPA SMCs. (*G* and *H*) ACE2 (*G*) and TMPRSS2 (*H*) concentrations in lung fibroblasts. Data are presented as mean ± SEM. *P < 0.05. NX = normoxia.

Local tissue hypoxia and inflammation are associated with elevated extracellular ATP concentrations (17, 31) and therefore could be pathological factors during SARS-CoV-2 infection. Thus, we sought to determine if the mRNA expression of ACE2 and TMPRSS2 is regulated by hypoxia and ATP in these lung vascular cells (cultured in normal medium). We found that hypoxia (3% O_2,_ 24 h) and extracellular ATP (100 μM, 24 h) in combination upregulated the expression of ACE2 and TMPRSS2 in HLMVECs (Figures 2A and 2B). We observed an increased trend (*P* = 0.078) of ACE2 expression and significantly increased TMPRSS2 expression in pericytes after exposure to hypoxia and ATP (Figures 2C and 2D). However, no significant changes of ACE2 and TMPRSS2 were observed in dPA SMCs (Figures 2E and 2F) or lung fibroblasts in response to hypoxia and ATP (Figures 2G and 2H).

Considering that additional SARS-CoV-2 binding and entry cofactors can promote viral binding and endothelial activation (4), we sought to determine if the mRNA expression of CD147 (basigin, a plasma membrane signaling receptor of the IgG superfamily and an extracellular matrix metalloproteinase inducer) and the cysteine proteases cathepsin B and L are regulated by hypoxia and ATP in HLMVECs. We found that ATP treatment modestly decreased mRNA expression of CD147. The combination of both stimuli increased the mRNA expression of cathepsin B but not cathepsin L (*see* Figure E1).

### SARS-CoV-2 Does Not Productively Infect Primary HLMVECs in Either Normoxic or Hypoxic Conditions

**Figure 3.**
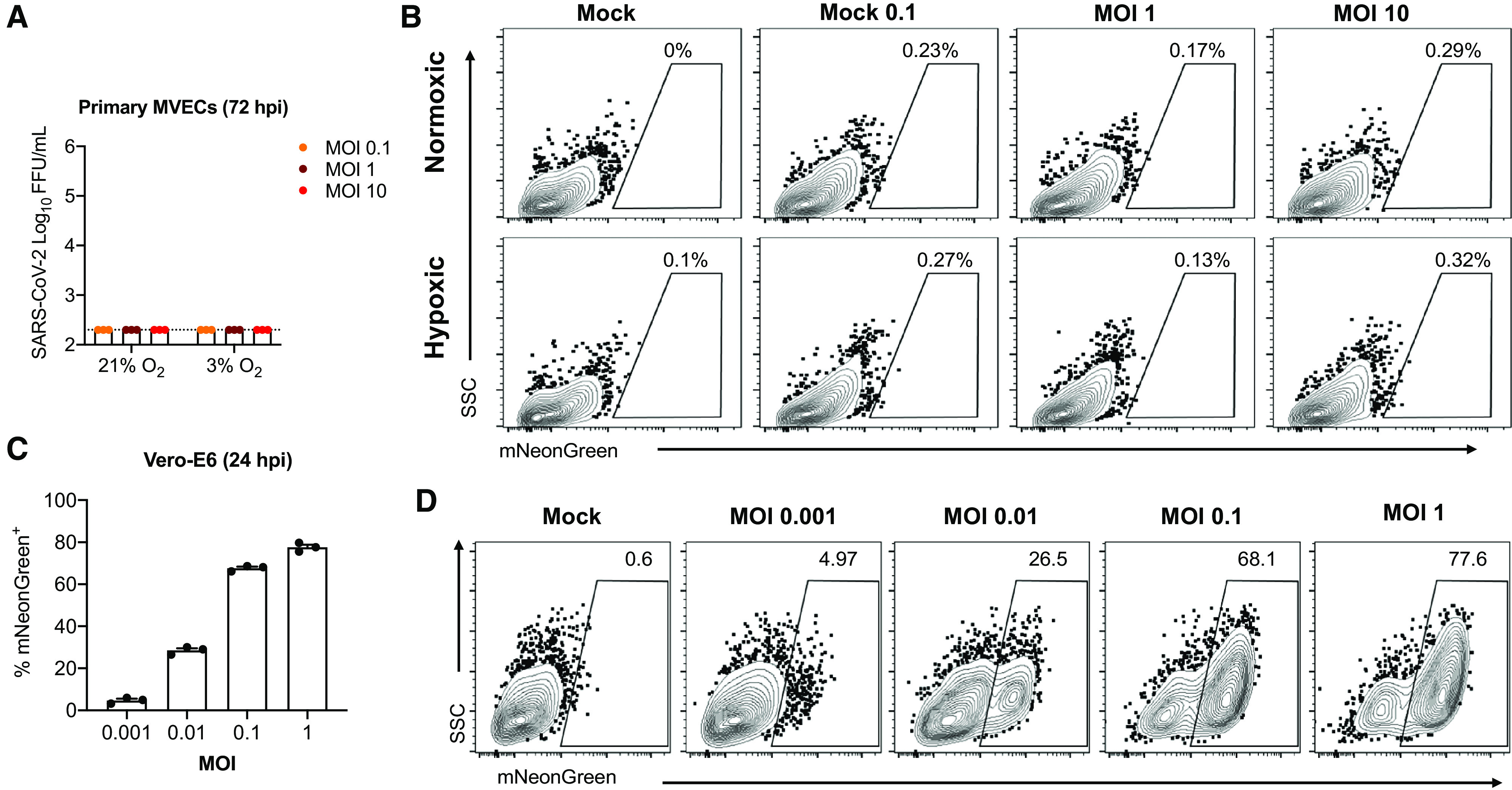
SARS-CoV-2 does not productively infect primary HLMVECs. (*A*) Measure of infectious virus in HLMVEC supernatants by FFA. (*B*) mNeonGreen reporter assay in infected HLMVECs. (*C*) Measure of infectious virus in susceptible Vero E6 cell supernatants by FFA. (*D*) mNeonGreen reporter assay in infected Vero E6 cells. *See* Methods for details. FFA = focus formation assay; FFU = fluorescence-forming units; hpi = hours postinfection; MOI = multiplicity of infection; MVEC = microvascular endothelial cell; SARS-CoV-2 = severe acute respiratory syndrome coronavirus 2; SSC = side scatter.

The low expression of ACE2 and TMPRSS2 in endothelial and other lung vascular cells raised questions regarding the susceptibility of these cells to SARS-CoV-2 infection. To test this, cells were cultured under either normoxia or hypoxia for 24 hours and then inoculated with SARS-CoV-2-mNG at a range of MOIs. At 72 hours after virus inoculation, cells were evaluated for mNG expression using flow cytometry, and cell culture supernatants were evaluated for the presence of infectious virus using an FFA. These analyses demonstrated that SARS-CoV-2 did not productively infect HLMVECs (Figures 3A and 3B) or other resident vascular cells such as pericytes, fibroblasts, or dPA SMCs, as well as additional human pulmonary microvascular ECs and pulmonary artery ECs (*see* Figure E2) compared with the SARS-CoV-2–susceptible cell line Vero E6 used as a positive control (Figures 3C and 3D).

### SARS-CoV-2 Genomic RNA Can Be Detected, without Evidence for Viral RNA Replication, in HLMVECs Exposed to the Virus

**Figure 4.**
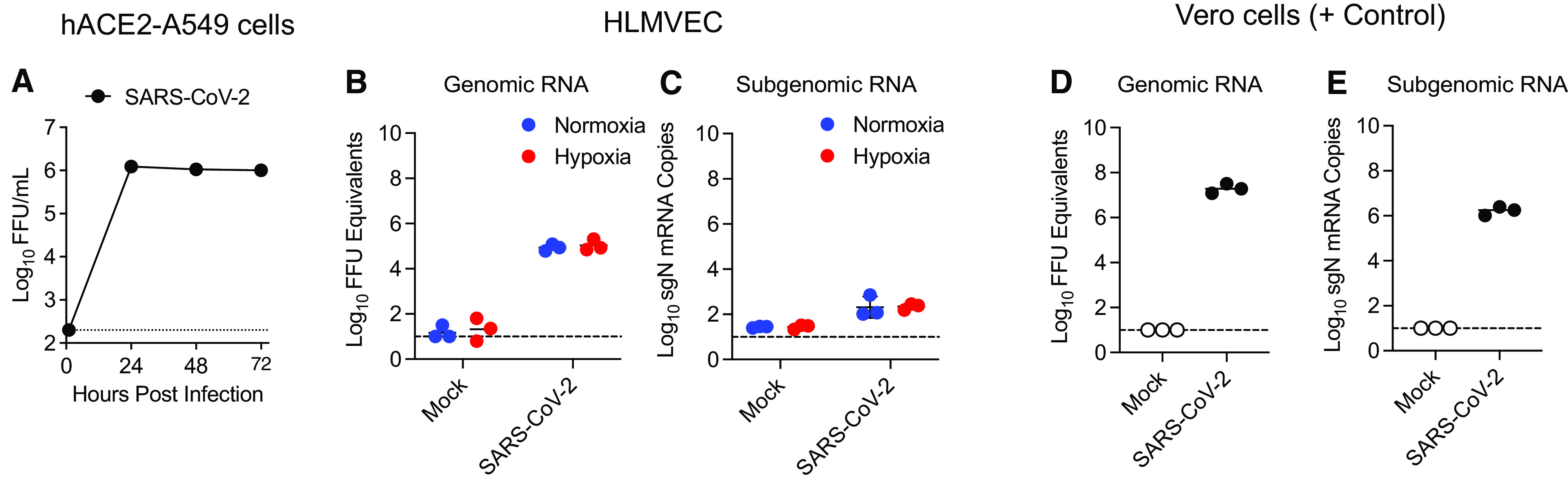
SARS-CoV-2 genomic RNA can be detected in HLMVECs after SARS-CoV-2 exposure. (*A*) Human ACE2 stably transfected A549 (hACE2-A549) cells were inoculated with SARS-CoV-2 (MOI = 0.5 FFU/cell). At 0 (input), 24, 48, and 72 hours postinoculation, infectious virus in cell culture supernatants was quantified using FFA. (*B* and *C*): HLMVECs were cultured under normoxic or hypoxic (3% O_2_) conditions for 24 hours. Cells were then stimulated for 24 hours with conditioned medium from mock-infected or SARS-CoV-2–infected hACE2-A549 cells (MOI = 1.5 FFU/cell). At 24 hours after conditioned medium treatment, viral genomic RNA (*B*) and N (nucleocapsid) subgenomic (sgN) mRNA (*C*) were quantified using qRT-PCR. (*D* and *E*) Susceptible and permissive Vero E6 cells were inoculated with SARS-CoV-2-mNeonGreen at an MOI of 0.001 FFU/cell in normoxia. At 24 hours postinoculation, viral genomic RNA (*D*) and sgN mRNA (*E*) were quantified using qRT-PCR.

As the pathogenic endothelial responses to SARS-CoV-2 have not been fully characterized, we investigated if viral entry into HLMVECs may occur even without productive virus replication, as shown above (Figure 3). For these experiments, we used conditioned medium from SARS-CoV-2–infected hACE2-A549 cells to mimic the likely sequence of events that occur in infected and hypoxemic lungs. As shown in Figure 4A, SARS-CoV-2 replicates efficiently in hACE2-A549 cells, with titers reaching 10^6^ FFU/ml by 24 hpi. Exposure of HLMVECs to conditioned medium collected from SARS-CoV-2–infected hACE2-A549 cells at 24 hpi (MOI = 1.5 FFU/cell) resulted in detectable SARS-CoV-2 genomic RNA but little to no detectable SARS-CoV-2 subgenomic RNA at 24 hpi under both normoxic (21% O_2_) and hypoxic (3% O_2_) conditions (Figures 4B and 4C). As a control for the viral RNA analysis, susceptible and permissive Vero E6 cells were inoculated with SARS-CoV-2 (MOI = 0.001 FFU/cell) in normoxia. At 24 hpi, both SARS-CoV-2 genomic RNA (Figure 4D) and subgenomic RNA (Figure 4E) were readily detected, indicative of active viral RNA replication. These data suggest that some viral uptake by ECs likely occurs without intracellular viral RNA replication.

### SARS-CoV-2 and Hypoxia Increased the Expression of Inflammatory Factors in HLMVECs

**Figure 5.**
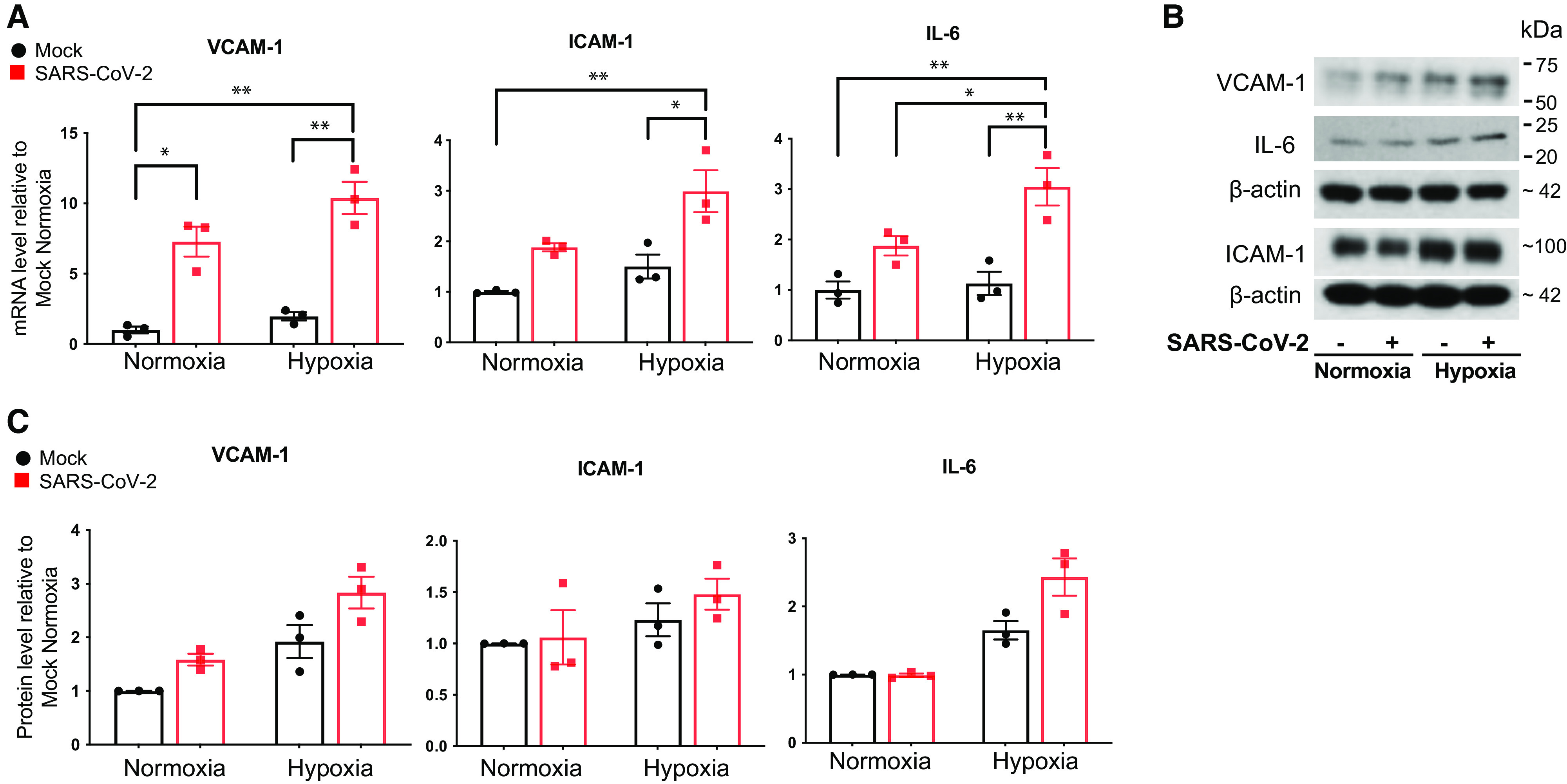
SARS-CoV-2 and hypoxia stimulate inflammatory responses in HLMVECs. HLMVEC were preexposed to normoxic or hypoxic (3% O_2_) conditions for 24 hours, followed by the addition of conditioned medium from mock-infected or SARS-CoV-2–infected hACE2-A549 cells for 24 hours. (*A*) Total RNA was isolated, and the mRNA concentrations of indicated inflammatory markers were determined using qRT-PCR analysis. Data are presented as mean ± SEM. *P < 0.05 and **P < 0.01. (*B*) Total cell lysates were collected and analyzed for the expression of indicated markers using western blot analysis, and the representative blots are presented. (*C*) Densitometry of the blots in *B* is presented (technical replicates). ICAM-1 = intercellular adhesion molecule 1.

Detection of SARS-CoV-2 genomic RNA in HLMVECs exposed to medium collected from SARS-CoV-2–infected hACE2-A549 cells led us to examine if pyroptosis and/or inflammatory signaling is activated in response to hypoxia and SARS-CoV-2–infected lung alveolar epithelial cells. Pyroptosis, an inflammatory form of cell death, has been reported in several cell types, including ECs in human diseases, including SARS-CoV-2 infection (14, 15, 32). More important, it was reported that the induction of pyroptosis might have an important role in EC injury, and it was proposed to contribute to the lung pathology observed in patients with COVID-19 (33). Therefore, we examined whether SARS-CoV-2 exposure induces pyroptosis in HLMVECs. As shown in Figure E3, exposure of HLMVECs to conditioned medium from SARS-CoV-2–infected hACE2-A549 cells under either normoxic or hypoxic conditions did not result in significant induction of classical pyroptosis markers, including gasdermin D, caspase-4, caspase-5, and HMGB1 (high mobility group box 1). However, we observed activation of caspase-1 (decreased trend of total caspase-1 and increased trend of cleaved caspase-1), suggesting that inflammasome activation may occur independent of pyroptotic cell death in response to SARS-CoV-2. We then examined the effect of the conditioned medium from SARS-CoV-2–infected hACE2-A549 cells on the expression of inflammatory markers and cytokine production in HLMVECs. We observed the upregulation of mRNA concentrations of adhesion proteins VCAM-1 (vascular cell adhesion molecule 1), ICAM-1 (intercellular adhesion molecule 1), and the proinflammatory cytokine IL-6 (interleukin 6) (Figure 5A). Moreover, hypoxia potentiated the effects of SARS-CoV-2–mediated expression of IL-6 (Figure 5A). Consistent with the findings at the mRNA level, we observed increased trends of VCAM-1, ICAM-1, and IL-6 protein concentrations in HLMVECs when exposed to SARS-CoV-2 and/or hypoxia (Figures 5B and 5C).

### Conditioned Medium from SARS-CoV-2–infected hACE2-A549 Cells and Hypoxia Upregulate the Expression of Complement in HLMVECs

**Figure 6.**
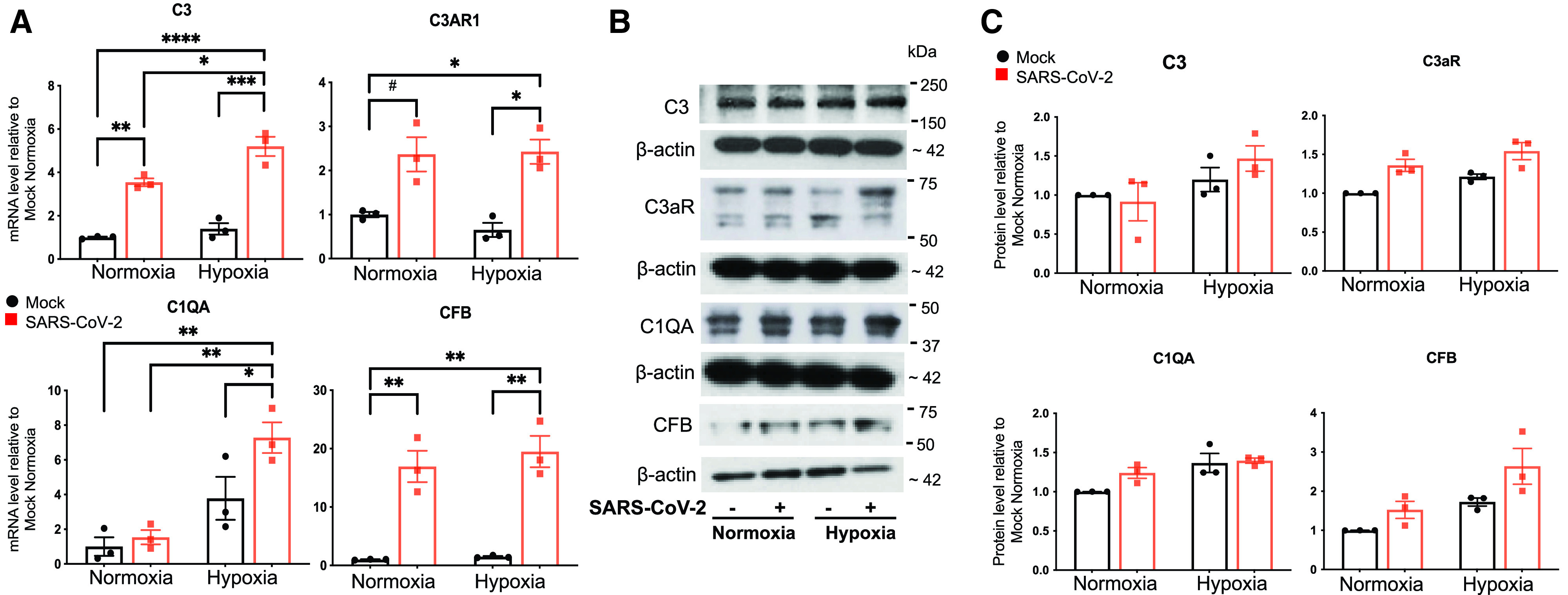
Effects of the conditioned medium of SARS-CoV-2–infected hACE2-A549 cells and hypoxia on the expression of complement proteins in HLMVECs. HLMVECs were preexposed to normoxic or hypoxic (3% O_2_) conditions for 24 hours, followed by the addition of conditioned medium from mock-infected or SARS-CoV-2–infected hACE2-A549 cells for 24 hours. (*A*) Total RNA was isolated, and mRNA concentrations of C3 (complement C3), C3AR1 (complement C3a receptor 1), C1QA (complement C1q A chain), and CFB (complement factor B) were determined using qRT-PCR analysis. Data are presented as mean ± SEM. *P < 0.05, **P < 0.01, ***P < 0.001, ****P < 0.0001, and ^#^*P* = 0.0508 (borderline significant). (*B*) Total cell lysates were collected and analyzed for the expression of indicated complement components using western blot analysis, and the representative blots are presented. (*C*) Densitometry of the blots in *B* is presented (technical replicates).

As complement deposition and hyperactivation have been associated with SARS-CoV-2–mediated endothelial damage, we sought to determine if complement system genes are upregulated in HLMVECs after exposure to conditioned medium from SARS-CoV-2–infected hACE2-A549 epithelial cells and whether this response is potentiated by hypoxia. HLMVECs were exposed to the conditioned medium of uninfected or infected hACE2-A549 cells and hypoxia (3% O_2_, 24 h). As shown in Figure 6A, the conditioned medium from SARS-CoV-2–infected hACE2-A549 cells upregulated the mRNA concentrations of C3 (complement C3), C3aR1 (complement C3a receptor 1), CFB (complement factor B), and C1QA (complement C1q A chain) in both normoxic and hypoxic conditions. A modest potentiating effect of hypoxia on the effects of conditioned media from SARS-CoV-2–infected cells was observed for C3 and C1QA expression. Consistent with the findings at the mRNA level, we observed increased trends of complement C3, C3aR, CFB, and C1QA protein concentrations in HLMVECs with SARS-CoV-2 infection and/or hypoxia exposure (Figures 6B and 6C).

## Discussion

The vascular pathology observed in COVID-19 remains a subject of intensive investigation (1, 34). In particular, the specific molecular mechanisms of SARS-CoV-2–mediated vascular damage in the context of systemic vascular inflammation and the activation of the complement system are not fully understood. As the lungs are the primary target organ of SARS-CoV-2 infection and exhibit signs of inflammatory vascular injury and local complement activation (26, 27), this study was undertaken to examine the susceptibility of lung vascular cells to SARS-CoV-2 infection and to determine whether the activation of a local endothelial complement system can be observed in response to viral exposure and be augmented by hypoxic conditions.

Histopathological and ultrastructural studies have demonstrated the presence of SARS-CoV-2 particles in the lungs and other organs (35), as well as in pulmonary, renal, and skin endothelium (3, 33, 36). COVID-19 tissue atlases have demonstrated that viral RNAs were enriched in mononuclear phagocytic and endothelial lung cells and multiple other cell types (37), indicating that SARS-CoV-2 cellular uptake may occur as a result of host cell responses in different cell types. It was demonstrated that engineered human capillary organoids were susceptible to infection with SARS-CoV-2, which was confirmed by the recovery of viral RNA from organoids postinfection (38). However, whether active SARS-CoV-2 infection occurs in ECs remains debatable, when the methods and approaches used to prove endothelial SARS-CoV-2 localization in tissue samples are considered (33, 39). As suggested by previous studies, the expression of the SARS-CoV-2 cell entry receptor ACE2 and protease TMPRSS2 positively correlates with cellular susceptibility to SARS-CoV-2 infection. Although the highest concentration of ACE2 was observed in epithelial cells (10), ACE2 expression was detected in ECs, SMCs, and pericytes (7, 40). Both ACE2 and TMPRSS2 are expressed by lung fibroblasts (41). In addition, expression of ACE2 was found in cardiac microvessels and cardiomyocytes, implicating SARS-CoV-2 infection of cardiac cells in the myocarditis and cardiovascular complications observed in some patients with COVID-19 (6, 42). However, relatively low expression concentrations of ACE2 and other entry factors in ECs may confer low susceptibility of the vascular endothelium to viral infection (4, 10, 11). Therefore, considering previous observations and the strong evidence of the involvement of the vascular system in the pathology of COVID-19, we examined the gene expression concentrations of ACE2 and TMPRSS2 and the susceptibility of different lung vascular cell types to SARS-CoV-2 infection. We did not find a direct SARS-CoV-2 permissive infection of HLMVECs, lung pericytes, fibroblasts, or SMCs, using assays to detect expression of a virus-encoded reporter protein (mNG) in infected cells, expression of subgenomic viral RNA, and production of extracellular infectious virus particles. A lack of productive infection of these cell types was concurrent with the low expression concentrations of ACE2, TMPRSS2, cathepsins B and L, and CD147. In addition, despite hypoxic upregulation of ACE2 and TMPRSS2 in HLMVECs, we did not observe any enhancing effect of hypoxia on SARS-CoV-2 infection, the condition that could prime lung EC activation state in COVID-19. These observations are in agreement with previous reports showing that low ACE2 expression and/or intracellular localization may explain why some cells cannot be productively infected by SARS-CoV-2 (12, 43). As suggested by recent studies, ACE2 glycosylation may be another factor that interferes with virus infectivity by preventing ACE2 binding to SARS-CoV-2 spike protein (44). EC glycocalyx is dynamically regulated in response to pathological conditions (45), so the role of ACE2 glycosylation in SARS-CoV-2 infection cannot be ruled out.

Recent reports indicate that ECs may be activated by SARS-CoV-2 and/or be susceptible to viral entry without productive replication (abortive infection). For example, SARS-CoV-2 spike protein alone can induce the impairment of EC physiology via downregulation of ACE2 expression and consequent inhibition of mitochondrial function (46). Comparative studies on various ECs have demonstrated that although most cell types are not susceptible to infection, SARS-CoV-2 spike protein was detected in coronary artery ECs in response to viral exposure (43). The presence of viral nucleocapsid protein in HLMVECs was demonstrated using fluorescent confocal microscopy, suggesting that pulmonary endothelium is permissive to abortive SARS-CoV-2 infection (13). In agreement with these reports, we demonstrated the presence of SARS-CoV-2 genomic RNA, but not subgenomic RNA, in HLMVECs, suggesting that these cells support some viral uptake without productive viral replication.

Pathological vascular responses to COVID-19 include morphological and structural alterations in microvascular ECs in the form of intussusceptive angiogenesis in the lungs (3), as well as endothelial hyperactivation, inflammation, and cell death via apoptosis and/or pyroptosis (14, 32, 33). Pyroptosis is characterized as cellular inflammatory and lytic cell death that occurs upon infection with intracellular pathogens and results in the release of several damage-associated molecular patterns, such as HMGB1 and inflammatory cytokines (15). SARS-CoV-2 was shown to trigger NLRP3 inflammasome and caspase-1–mediated cytokine release and pyroptosis in several cell types, including monocytes and hematopoietic stem or progenitor cells (14, 15, 32). However, the pathological responses of ECs to SARS-CoV-2 exposure remain incompletely understood. Detection of SARS-CoV-2 genomic RNA in HLMVECs led us to examine if pyroptosis and/or inflammatory signaling are activated in response to viral uptake. Our data did not provide evidence of SARS-CoV-2–mediated pyroptosis, as shown by the lack of gasdermin D, caspase-4 and caspase-5 cleavage, and a lack of upregulation of HMGB expression. Considering that each cell type has specific pyroptotic markers that may not overlap with classical markers (e.g., gasdermin-D) and the lack of comprehensive experiments exploring pyroptosis pathways in the present study, whether SARS-CoV-2 exposure induces pyroptosis in HLMVECs will need to be further explored. On the other hand, SARS-CoV-2–mediated caspase-1 cleavage suggests the activation of the NLRP3 inflammasome and caspase-1–mediated inflammatory responses in HLMVECs.

A lack of EC susceptibility to productive SARS-CoV-2 infection may suggest a biological significance of vascular endothelium as a mucosal barrier, preventing viral dissemination into systemic circulation and organ damage. Nevertheless, indirect endothelial activation by soluble paracrine factors released by infected and damaged cells (17) may play a pathophysiological role in COVID-19. Alveolar hypoxemia and airway epithelial cell damage in response to SARS-CoV-2 infection result in a proinflammatory environment in the lung epithelial alveolocapillary interface (1, 47). Release of viral particles from damaged epithelial cells to the epithelial–endothelial interface also contributes to endothelial activation, perhaps via alternative, ACE2-independent mechanism(s). Therefore, we assessed the possibility that endothelial activation may result from factors released by infected epithelial cells and examined whether hypoxia might sensitize SARS-CoV-2–mediated proinflammatory responses and complement activation in HLMVECs. Our findings showed that conditioned media from productively infected A549 cells overexpressing ACE2 and hypoxia upregulated the expression of the adhesion molecules VCAM-1 and ICAM-1, as well as the inflammatory cytokine IL-6. These responses were, to various extents, potentiated by hypoxia, suggesting that in a hypoxic lung environment, endothelial inflammation and hyperpermeability may contribute to vascular dysfunction in COVID-19. These findings are consistent with previous studies showing that severe COVID-19 lung disease is influenced by a temporal expression of various pathological signaling pathways, and in the early stages, there is a strong inflammatory response leading to subsequent tissue hypoxia and microthrombosis (48).

The complement system is an important component of the innate immune system and plays a role in a variety of pulmonary conditions, including SARS-CoV-2 infection, by mediating vascular inflammatory and thrombotic responses (19–21, 23, 49). Clinical data have demonstrated evidence of increased C3a and C5a complement production, “cytokine storm,” proinflammatory macrophage accumulation, and cellular apoptosis in COVID-19 and other lung diseases (20, 49). Previous work in our laboratory demonstrated that activation of the complement cascade was consistently observed in a perivascular-specific manner in human PAH and laboratory animal models of hypoxic pulmonary hypertension (27). However, it remained unclear if endothelial complement activation is associated with endothelial inflammatory responses in SARS-CoV-2 infection and if it could be enhanced by hypoxic signaling (50). As pulmonary ECs can be indirectly activated by soluble paracrine factors (17), we evaluated the effects of hypoxia and extracellular ATP (a proinflammatory and damage-associated molecular pattern molecule) on the expression of complement components in HLMVECs. We found the upregulation of complement components, including C3, C3aR1, CFB, and C1QA, in response to hypoxia and/or ATP, indicating that local complement activation may be a consequence of the SARS-CoV-2–mediated proinflammatory microenvironment in the lungs. Moreover, our experiments with conditioned medium of SARS-CoV-2–infected hCE2-A549 cells and hypoxia further confirmed the upregulation of complement proteins C3, C3aR1, C1QA, and CFB in HLMVECs, also demonstrating a modest potentiating effect of hypoxia on the expression of C3 and C1QA. Thus, it can be postulated, that alveolar SARS-CoV-2 infection and subsequent hypoxia could be pathogenic factors in COVID-19 contributing to the production of autocrine and/or paracrine factors that mediate local complement deposition and endothelial activation.

### Conclusions

Taken together, our observations provide evidence that during SARS-CoV-2 infection, hypoxia-induced inflammation and activation of the local complement system are potentially involved in the endothelial and vascular dysfunction in COVID-19. We confirmed previously reported findings on a lack of endothelial susceptibility to SARS-CoV-2 productive infection and demonstrated that hypoxia and SARS-CoV-2 may act synergistically to stimulate inflammatory responses and complement activation in HLMVECs. The results of our study may suggest that therapeutic strategies directed at strengthening of endothelial barrier, eliminating vascular inflammation, and complement activation could be helpful in ameliorating endothelial injury and cardiovascular complications in patients with COVID-19.
